# Synaptic Mechanisms of Blast-Induced Brain Injury

**DOI:** 10.3389/fneur.2016.00002

**Published:** 2016-01-21

**Authors:** Andrzej Przekwas, Mahadevabharath R. Somayaji, Raj K. Gupta

**Affiliations:** ^1^Computational Medicine and Biology Division, CFD Research Corporation, Huntsville, AL, USA; ^2^Department of Defense Blast Injury Research Program Coordinating Office, U.S. Army Medical Research and Materiel Command, Fort Detrick, MD, USA

**Keywords:** synapse, TBI, neuro-axonal, blast wave injury, mechanobiology, DAI

## Abstract

Blast wave-induced traumatic brain injury (TBI) is one of the most common injuries to military personnel. Brain tissue compression/tension due to blast-induced cranial deformations and shear waves due to head rotation may generate diffuse micro-damage to neuro-axonal structures and trigger a cascade of neurobiological events culminating in cognitive and neurodegenerative disorders. Although diffuse axonal injury is regarded as a signature wound of mild TBI (mTBI), blast loads may also cause synaptic injury wherein neuronal synapses are stretched and sheared. This synaptic injury may result in temporary disconnect of the neural circuitry and transient loss in neuronal communication. We hypothesize that mTBI symptoms such as loss of consciousness or dizziness, which start immediately after the insult, could be attributed to synaptic injury. Although empirical evidence is beginning to emerge; the detailed mechanisms underlying synaptic injury are still elusive. Coordinated *in vitro–in vivo* experiments and mathematical modeling studies can shed light into the synaptic injury mechanisms and their role in the potentiation of mTBI symptoms.

## Introduction

Traumatic brain injury (TBI) has become the signature wound of military operations since service members are frequently exposed to roadside bombs and explosions. The majority of combat-related TBI cases are categorized as mild, primarily based on event history and post-injury assessment of behavioral and cognitive symptoms. Mild TBI (mTBI) typically induces a variety of heterogeneous symptoms, including concentration problems, blurred vision, irritability, headaches, sleep disorders, and depression. The symptoms may also be associated with cognitive and neurodegenerative disorders, such as post-traumatic stress disorder (PTSD) and chronic traumatic encephalopathy (CTE) (1–3). In spite of its importance and many years of research, current understanding of the primary (biomechanics) and secondary (neurobiology) brain injury mechanisms is limited. Moreover, the link between primary injury biomechanics, the neurobiology of secondary injury and repair, and the resultant neuropsychological and neurodegenerative outcomes remain elusive.

In the last few years, several hypotheses have been proposed to explain the mechanism of blast-induced primary brain injury. These include elastic and shear waves in brain, brain rotation relative to cranium, brain–skull contact, cavitation, electromagnetic pulse, axonal damage, micro hemorrhage, vascular elastic waves propagating from the blast loaded thorax to the brain, damage to the bridging veins, and others (4–9). In contrast to focal injury, diffuse axonal injury (DAI) occurs in a widespread area and is a common pathology observed in blunt and blast-induced TBI (10–13). The primary micro-damage associated with it is manifested by impairment to neurofilament units of the axonal cytoskeleton, loss of membrane integrity, and Wallerian-type axonal degeneration (14–17). Although the mechanism of DAI is thought to originate from acceleration, deceleration, and rotational forces, its pathogenesis is largely attributed to the onset of secondary injury cascade. Non-invasive brain imaging studies of humans exposed to blasts with mTBI symptoms using advanced diffusion tensor MRI (DT-MRI) have confirmed DAI signatures (12).

In the acute injury phase, diffuse structural changes to the synaptic clefts and postsynaptic densities (PSDs) cause temporary loss of neural circuit connectivity. This primary micro-damage initiates a cascade of biophysical and neurochemical events, lasting from minutes to hours, resulting in either axonal and synaptic repair or permanent damage (18, 19). Moreover, some mTBI-relevant cognitive deficits, such as loss of consciousness (LOC) or dizziness, start immediately after the insult while others, including headaches, fatigue, depression, learning/memory deficits, and neurodegeneration, take a longer time to evolve. However, it is difficult to attribute the temporal diversity of injury responses only to DAI. We posit that biomechanical micro-damage to axons as well as neuronal synapses and dendritic spines play a major role in the etiology of mTBI. Better understanding of the dynamics of diffuse synaptic injury may offer a window of opportunity in which an appropriate treatment may modify an imbalance between post-injury excitatory and inhibitory processes.

## Neurotransmission and Neuroplasticity

The human brain is organized as a highly interconnected structural network of neurons, glia, and supporting cells responsible for cognitive and physiological information processing. The structural integrity of brain neurons and glia is maintained by a complex network of extracellular matrix. It is estimated that the adult human brain contains ~10^11^ neurons, each of them equipped with ~10^4^ synapses (20, 21). This huge connectome of ~10^15^ interconnects undergoes continuous remodeling in response to a variety of stimuli, a process termed as neuroplasticity (22). These changes include structural remodeling of presynaptic terminals, PSDs, dendritic spines, adhesion molecules, and the surrounding glial cells. The magnitude and direction of these changes depend on the duration and frequency of presynaptic stimulation. Synaptic plasticity, a type of neuroplasticity, is the activity-dependent change in the synaptic strength and efficacy and forms the neurochemical basis of learning and memory. Below the level of the synapse, the physiological neuroplasticity involves complex mechanisms of gene expression, protein synthesis, receptor trafficking to and on the dendritic membrane, recruitment of new receptors, remodeling of synaptic adhesion proteins or even removal of some, and formation of new, synaptic clefts. It has been observed in magnetic resonance elastography brain imaging that brain neuronal structures are continuously modulated by physiological micromechanical loadings originating from intracranial pressure pulsations due to cardiac and respiratory rhythms as well as head movement (23). It is believed that these micro mechano-biological effects and their interaction with neurochemical pathways are essential for proper brain function and neuroprotection (24–28). On the other hand, non-physiological, high speed loadings, such as in accidental head impacts or blast waves, may cause acute injuries to neuronal microstructures, such as axons, dendritic spines, and synapses, with neuropathological implications.

**Figure 1:**
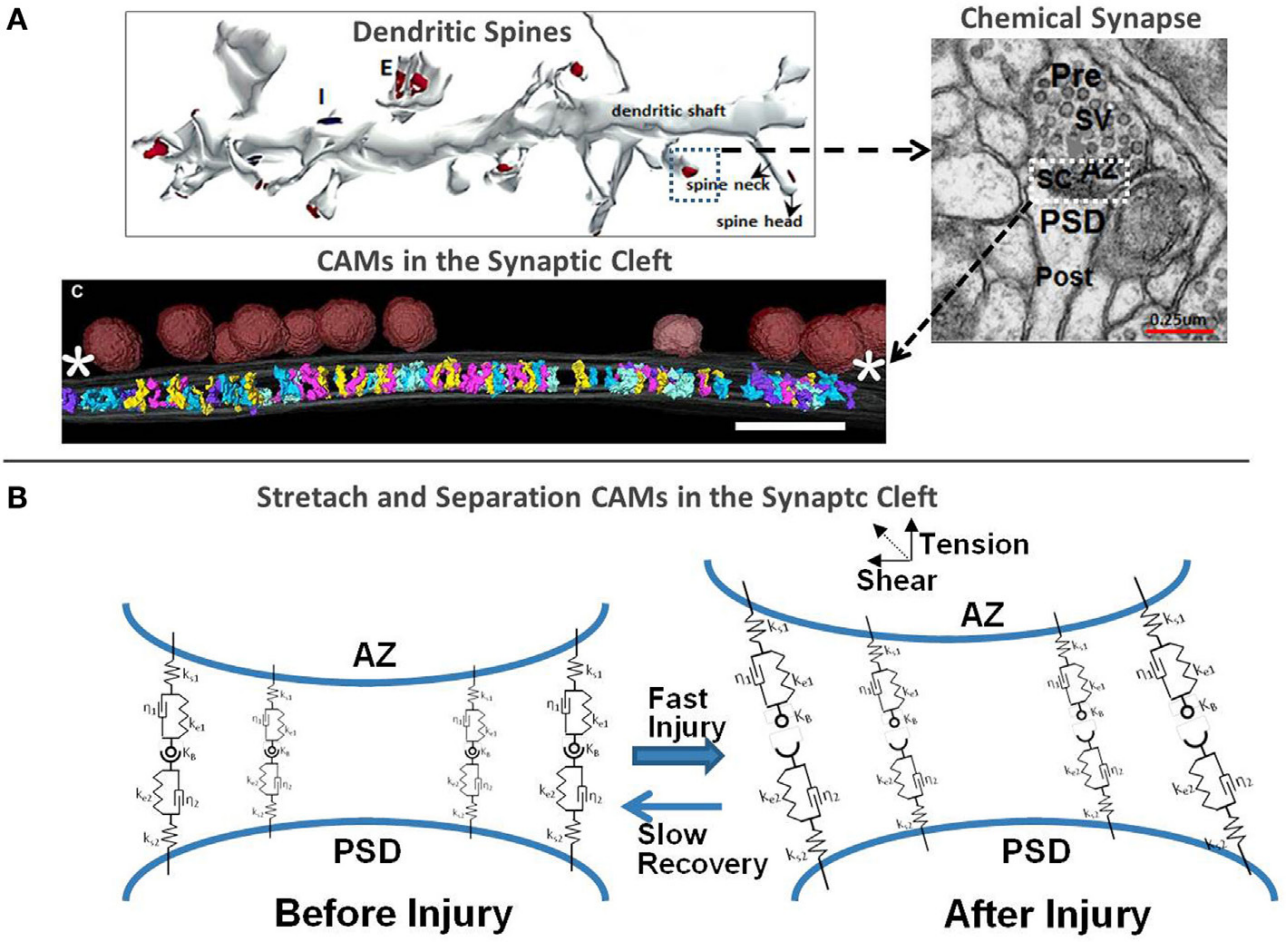
**(A)** 3D rendering of a segment of the dendritic shaft with excitatory and inhibitory chemical synapses, morphology of the synaptic cleft (30, 31) and 3D rendering of CAMs in the synaptic cleft [adapted from High et al. (32)] and **(B)** biomechanical representation of the cleft scaffolding proteins using spring-damper network and injury-induced perturbations.

Figure 1 illustrates the distribution of neuronal synapses, neurotransmission, and our perspectives on synaptic injury mechanisms. Each neuron is connected to other neurons via large number of excitatory (E) and inhibitory (I) chemical synapses located on the dendritic arbor and cell body. The chemical synapse is a highly organized structure consisting of a presynaptic terminal juxtaposed across a postsynaptic button on top of a dendritic shaft and is often surrounded by astrocytic processes (astrocytes not shown in Figure 1). The action potential arriving at the presynaptic terminal causes the release of a neurotransmitter (NT) into the synaptic cleft: glutamate (Glu), norepinephrine, etc., at E-synapses and γ-aminobutyric acid (GABA) and serotonin at I-synapses. The NT diffuses through this narrow cleft and binds to ion channels and receptors on the postsynaptic neuronal membrane. The influx of ions alters the postsynaptic voltage causing either a depolarization (E signal) or hyperpolarization (I signal) in the dendritic branch of the postsynaptic neuron. The number of E and I signals that a single neuron receives dictates its excitability and function. In other words, the likelihood of firing an action potential by the receiving neuron depends on the number of E and I synaptic potentials and the somatic summation. In the case of glutamatergic synapses, the small size of the synaptic cleft (~2 attoliters) facilitates rapid rise of Glu concentration in the cleft, rapid binding to PSD receptors, and fast clearance. The small size and precise organization of the synapse facilitate high frequency neurotransmission with rapid buildup of Glu concentrations of 1–5 mM in the cleft post-stimulation, followed by a fast clearance by Glu transporters, in <1 ms (29). Mechanical extension or shear deformation of synapses post-injury may have detrimental effects on neurotransmission.

## Morphology of Dendritic Spines, E- and I-Synapses

In the central nervous system, the majority of E- and I-synapses differ in their location, composition, structure, and function (33, 34). The E-synapses are localized on dendritic spines, which are terminated with dense organelles (PSDs), hosting hundreds of scaffolding and signaling molecules involved in neurotransmission and synaptic plasticity. Most GABAergic I-synapses, on the other hand, are formed directly on dendritic shafts, as well as on the soma and proximal axonal regions. Endogenous regulatory mechanisms precisely maintain the E/I balance and any acute or chronic E/I perturbations may be responsible for various pathologies, including epilepsy, depression, and other disorders (34–37). From a mechanical perspective, the postsynaptic dendritic spine of an E synapse looks like a cantilever beam having a big heavy head with weak thin neck. The dendritic head is typically ~0.5–2 μm in diameter and is connected to the parent dendrite by a thin spine of ~0.04–1 μm in length (34). We conjecture that E-synapses located on tall dendritic spines could be more vulnerable to mechanical damage during tension and shear loadings than I-synapses positioned at dendritic roots and soma.

Another mechanically sensitive synaptic structure is the synaptic cleft, with a typical dimension of 15–25 nm height and ~300 nm diameter. It is filled with structural proteins as well as cell adhesion molecules (CAMs) that hold pre- and postsynaptic membranes together at the appropriate juxtaposition. Some of them, e.g., neurexins (NXs), neuroligins (NLs), SynCAMs, and integrins, localize at the center of the synapse, whereas others, such as Cadherins, reside at the synaptic periphery. However, transcleft elements in both E- and I-synapses typically avoid places where synaptic vesicles attach to the presynaptic membrane (32). The extracellular domains of these CAMs, protruding from the opposite sides of the synaptic terminals, are “sticky,” and are bound by hydrophobic forces to each other in either homophilic (e.g., Cadherins) or heterophilic (e.g., presynaptic NXs and postsynaptic NLs) combination and mechanically maintain the synaptic structure. Their cytoplasmic domains bind to and mechanically modulate adaptor proteins that organize the synaptic structure and function as well as the morphologies of the presynaptic active zone (AZ) and PSD. Some CAMs, such as NXs and NLs, and cadherins require extracellular calcium to maintain their elasticity and binding affinity. At physiological conditions, CAMs behave as elastic springs and are under tension force (~10–20 pN) that makes them “longer” than in crystallographic dimensions. Calcium shifts from interstitial to intracellular spaces following mechanical injury are often considered a major contributing factor to metabolic, excitatory, and apoptotic pathways in TBI (38). It is also possible that reduced synaptic calcium concentration can affect binding affinity and mechanical stiffness of Ca-dependent binding of CAMs. In the absence of depleted intra-synaptic calcium, cadherins lose their elastic strength and behave as “loose and weak rope” (39). For example, NX-1β and NL1 form a strong transsynaptic heterotetramer only in the presence of 1–3 μM free Ca^2+^ (40). Atomic force microscopy measurements at slow loading rates have shown that rupture forces for CAMs range between 50 and 70 pN (41–43). Although astrocytes are not directly structurally linked to the synaptic cleft, astrocytic processes are in close apposition to the synaptic structures, form tripartite synapses and mechanically interact with them through the ECM. They also act as diffusion barriers to NT spillover, facilitate NT uptake, and provide local metabolic support. To better articulate the proposed hypothesis, in the following, we will focus only on bipartite synapses.

## Do Blast-Induced Forces Affect Macroscopic Brain Biomechanics and Neuronal Structures?

At the macroscopic scale, there are multiple pathways for blast-induced forces and energy content to impact the brain. Previous computational and experimental works have shown that the cranial bone is a good transmitter of elastic waves to the CSF and brain with little attenuation below 10^4^–10^5^ Hz (44, 45). During blast wave loading, the brain experiences two types of biomechanical events: (1) rapid elastic skull deformation causing compression/tension stress waves, which later dissipate as shear waves and (2) delayed head movement causing brain rotation relative to the skull, which then generates shear waves within the brain. Specifically, compression/tension waves propagate in the brain with the speed of sound (~1500 m/s), transform into shear waves and last only few milliseconds. Shear waves, on the other hand, are orders of magnitude slower (~10 m/s), dissipate due to viscous action and persist longer, up to hundreds of milliseconds (45). It has been documented that angular accelerations of the brain often lead to DAI, contusion, and acute subdural hematomas (14, 17, 46–53). Experimental tests on human volunteers (54) and cadavers (55, 56) have shown brain translation magnitudes of 4–5 mm and rotation of ±5° for ~300 ms at low-severity impacts in the sagittal plane. The macroscopic brain biomechanics is expected to be absorbed/dissipated at the cellular scales for potentiation of cellular injury.

At the microscopic scale, the brain tissue behaves as a heterogeneous non-linear viscoelastic material with multiple strain rates (57). All cells in the brain experience continuous mechanical forces from normal head movement and from intracranial and intracellular hydrodynamic and osmotic pressures, yet maintain their function. However, higher mechanical loads may cause inelastic structural damage to load bearing microstructures. For instance, the cantilever dendritic spines may undergo structural alterations when exposed to blast loads. Because the brain tissue is inhomogeneous, these strains concentrate at the micro-interfaces with impedance mismatch (e.g., disparate densities, morphologies) in the brain (7). *In vitro* and *in vivo* experiments show that tension and shear strains are much more damaging to the tissue than compressive strains (58). Intuitively, it can be explained that the surrounding water resists the compression and supports tissue structure while the tensile or shear force directly disrupts weaker (hydrogen, van der Waals) and stronger (covalent, ionic) bonds at the molecular level.

The rate of strain applied to viscoelastic brain tissue microstructures is also very important. At low strain rates, the tissue/cells are very ductile and can recover without damage from relatively large deformations. For example, experiments on human volunteers experiencing mild linear accelerations of ~1.5 G and angular accelerations of 120–140 rad s^−2^ show that significant regions of the brain exhibit 5–7% elongation strain (54, 59) and do not cause injury. However, at faster loads, the brain tissue becomes brittle and susceptible to micro-damage to brain cells/organelles, such as axons, synapses, vascular endothelium, membranes, cytoskeleton, ion channels, and other microstructures (14). This biomechanical “primary injury” lasting for a few milliseconds initiates a cascade of secondary injury (neurobiology) and recovery pathways lasting hours, days, and in some cases life times. Depending on the severity of the insult, some of the brain cells will rapidly transition to apoptosis and necrosis, while other injured cells and organelles may undergo a long lasting recovery process.

## Perspectives on Synaptic Injury in Blast-Induced mTBI

Synaptic injury mechanisms are largely unknown and have only recently begun to attract interest of neuroscientists partially because of experimental challenges at such small length and timescales (9, 25, 60–62). Recent experimental analyses have shown that the synaptic loss may be the secondary effects of DAI as a result of axonal fiber loss and synaptic terminal degeneration (63). *In vitro* experiments applying magnetic tweezer forces on neuronal structures have shown that mechanical damage to integrins, and potentially other CAMs, may be an important mechanism underlying the initiation of cell and sub-cellular injuries ultimately responsible for the diffuse axonal and synaptic pathology (64). Mechanical damage to neuronal micro/nano-structures, such as CAMs, cytoskeleton, membranes, and ion channels, is strain rate dependent. In sports and automobile related head injuries, the damage is caused by rotation-induced shear waves with large strains and typical strain rates of 100 s^−1^. Blast TBI involves very fast compression–tension wave followed by fast but slower shear waves with potentially smaller strain but much higher strain rates of the order of 1000 s^−1^ (65). The high strain rate of viscoelastic damage to neuronal micro/nano-structures may be more important in blast wave TBI, while slower but larger strains may be responsible for blunt and inertial TBI. It is also possible that the synaptic injury may be present in all types on mTBI. The detailed role of synaptic injury in blast and blunt loading patterns remains to be elucidated.

Experimental study of synaptic injury mechanisms is challenging. To date, it has been observed only in *in vitro* neuronal cultures subjected to a mechanical stretch (66, 67) and in brain slices of rodents exposed to shock waves (68, 69). Further studies of blast-induced synaptic injury mechanisms would be required using well-characterized blast waves or shock tubes for both *in vitro* cell/slice cultures (16, 65, 70) and *in vivo* animals (71, 72). Mechanical tension and shear waves may cause temporary disconnects and micro-damage of CAMs, synapses, and dendritic spines, which in turn may be manifested as temporary cognitive impairment (62, 67, 73). Moreover, reduced concentration of NTs and calcium in the deformed synapse may alter the connectivity of CAMs. As mentioned above, lower synaptic Ca^2+^ concentration reduces the elasticity of cadherins and diminishes the *de novo* hydrogen bond formation and would not allow cadherins to reassociate after injury. At the same time, it is also likely that a large number of mechanically deformed synapses in mTBI may self-restore by hydrophobic, electrokinetic, and other biophysical mechanisms due to other CAMs (39, 74).

Figure 1B schematically shows the structural response of CAMs to mechanical forces, which may cause their separation. Rapid tension or shear loads may cause separation of pre- and postsynaptic membranes, loss of contact between CAMs, plasma membrane mechanoporation and rearrangement of the cytoskeleton and scaffolding in the PSD and spinal neck. If the initial deformation is subcritical in the context of sufficient “healing” time, the CAMs may be able to reconnect and reestablish synaptic connection. This phenomenon may be one aspect of the neuro-recovery mechanisms. Transmembrane proteins, such as integrins and connexins, are responsible for structural reinforcement and alignment of both neuronal chemical synapses and electrical gap junctions. They also modulate a variety of intracellular pathways that are activated with the exertion of mechanical force on the integrin. It is likely that an inelastic damage to CAMs, integrins, and other focal adhesion molecules in brain injury affects not only the synaptic morphology but also the conductance of the ion channels and cytoskeleton remodeling. Recent experimental observations show that integrin-mediated activation of Rho may be a contributor to the DAI in mTBI (75) and suggest that a similar mechanism may be involved in synaptic injury as well (64). It is also possible that mechanical damage to N-cadherin–catenin complex, which stabilizes the cytoskeleton through Rho-family GTPases, may cause postsynaptic actomyosin contraction in dendritic spines and probable loss of excitatory synapses (76), similar to that observed in axon growth inhibition and retraction experiments (77).

**Figure 2:**
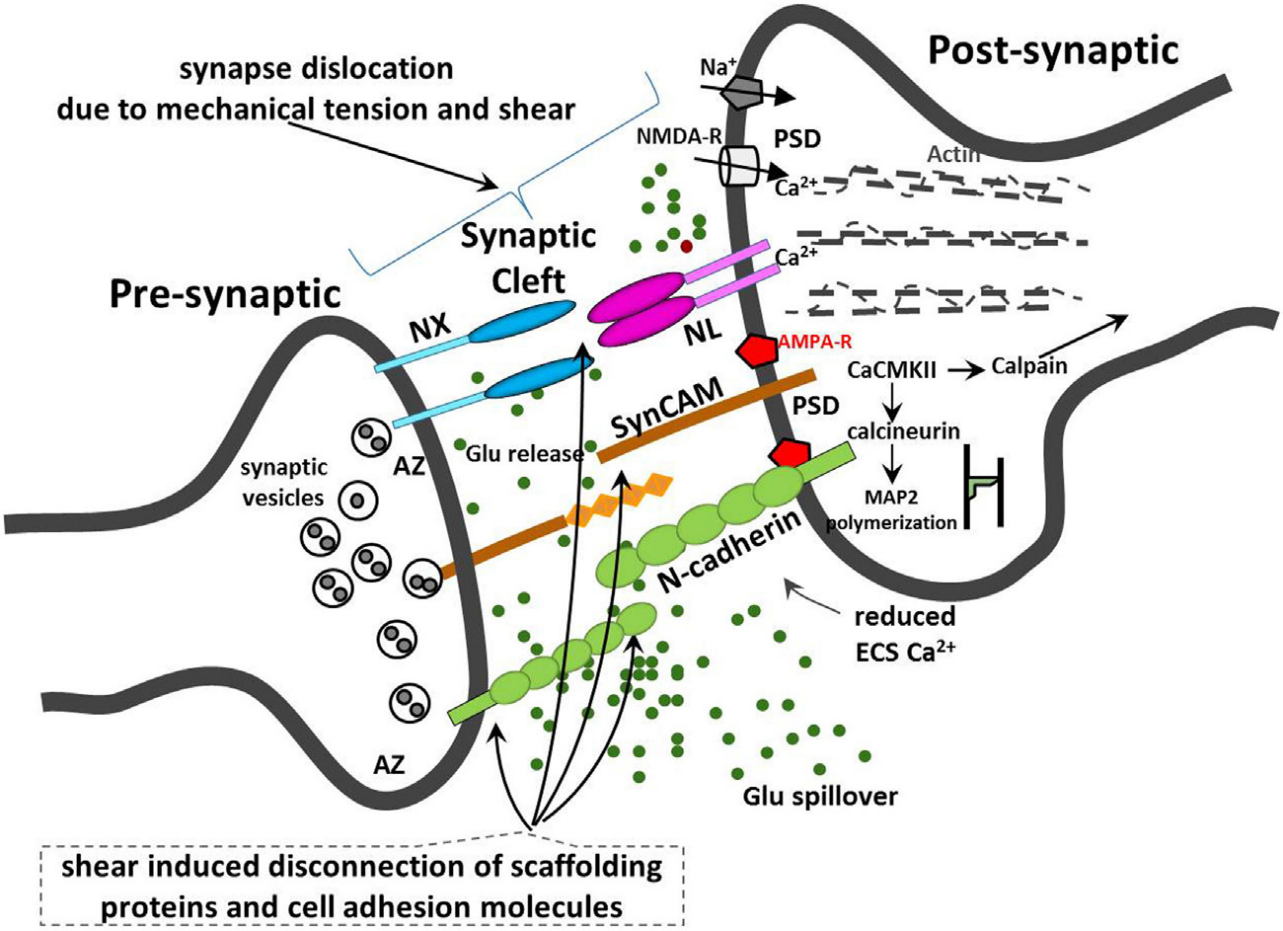
**Illustration of synaptic injury due to mechanical tension and shear and disruption to neuronal synaptic CAMs**.

The mechanical stretch or shear of the synaptic cleft will also alter the cleft volume, NT concentration, and diffusion distance and delay NT clearance. For instance, small changes in synaptic cleft height and geometry could retard Glu clearance and affect ion flux kinetics through ion channel, while higher Glu concentrations in the cleft may cause prolonged depolarization and excitotoxicity in postsynaptic neurons. Adverse neuroplasticity due to unnatural changes in synaptic morphology may affect LTP and/or LTD. Partial transient mechanoporation of dendritic and spinal membranes may cause ion leakage, affect the dynamics of de- and repolarization, induce calcium-mediated excitotoxicity, and cause increased energy demands and energetic “exhaustion” and oxidative stress. Additionally, Glu may also diffuse to a neighboring synapse and may inadvertently activate their receptors, illustrated in Figure 2.

## Conclusion and Future Direction

It is now clear that neural synapses transmit not only biochemical and electrophysiological information but also communicate using mechanical signals. Synaptic CAMs provide direct mechanical link between presynaptic vesicular release machinery and postsynaptic cytoskeletal and molecular organization. Composition, structural and elastic properties as well as intra-synaptic distribution of CAMs may be responsible for mechanical injury and repair pathways such as those involved in CTE but may also be implicated in developmental, cognitive, and neurodegenerative diseases, including autism (78), chronic stress and depression (79, 80), Alzheimer’s disease (81), schizophrenia (82), and other diseases (83, 84). Mechanobiology of axo-glial CAMs may be also implicated in the damage of myelinated axons in response to mTBI. Mechanical damage to axo-glial CAMs in response to TBI and the subsequent de/re-myelination are yet to be explored. Emerging evidence suggests that axo-glial CAMs, including NXs, NLs, and Nectin-like molecules, located in the narrow gap between the axonal membrane and the surrounding myelin sheath, are responsible for axon myelination and the so called “white matter plasticity” (85, 86). Finally, neuronal CAMs may be a promising pharmacological target for modulating the synaptic “connectome,” impaired in brain disorders and neurotrauma (87).

Coordinated *in vitro*–*in vivo* experiments and mathematical modeling studies should be conducted to shed light into the synaptic injury mechanisms and to determine whether the diffuse synaptic injury plays a prominent etiological role in mTBI. From a modeler’s perspective, it would be beneficial to collect *in vitro* and *in vivo* experimental data of geometry, morphology, and electrophysiology of a synaptic structure at various times post-injury. State-of-the-art fixing or high-pressure freezing, tomography, and electron microscopy of ultrathin sliced cell or tissue cultures can reveal the internal structure of synapses in exceptional 3D spatial resolution (32, 88). These techniques could be used to analyze the morphology of the synaptic ultrastructure post-injury. Less precise but equally helpful experiments could use super-resolution fluorescence microscopy techniques, such as STED or RESOLFT microscopy, to obtain time resolved synaptic remodeling data (89). Experimental elasto-mechanics studies of synaptic CAMs for various strain rates could provide not only the insight into their mechanobiology but also on the elasto-dynamic constants and damage thresholds, relevant for future mathematical models of synaptic injury. Better understanding of the role of CAMs in synaptic and axo-glial injury will require “animal models” that can be “molecularly engineered.” Genetically manipulated *Drosophila* and mouse models have been already developed and used for TBI research (62, 90, 91). Both models should be further pursued and complemented with the corresponding computational models to expedite the development of new treatments, diagnostics, and protective measures in blast-related neurotrauma.

## Author Contributions

AJP and MRS: conceived and drafted the manuscript (equal contribution). RKG: offered technical insights and reviewed the manuscript.

## Disclaimer

The views expressed in this paper are those of the authors and may not necessarily be endorsed by the U.S. Army or U.S. Department of Defense.

## Conflict of Interest Statement

The authors declare that the research was conducted in the absence of any commercial or financial relationships that could be construed as a potential conflict of interest.
